# Comparative Analysis of the Effects of Neurotrophic Factors CDNF and GDNF in a Nonhuman Primate Model of Parkinson’s Disease

**DOI:** 10.1371/journal.pone.0149776

**Published:** 2016-02-22

**Authors:** Enrique Garea-Rodríguez, Ave Eesmaa, Päivi Lindholm, Christina Schlumbohm, Jessica König, Birgit Meller, Kerstin Krieglstein, Gunther Helms, Mart Saarma, Eberhard Fuchs

**Affiliations:** 1 Clinical Neurobiology Laboratory, German Primate Center, Göttingen, Germany; 2 Department of Neuroanatomy Institute of Anatomy and Cell Biology, Albert-Ludwigs-University Freiburg, Freiburg, Germany; 3 Center for Molecular Physiology of the Brain (CMPB), University of Göttingen, Göttingen, Germany; 4 Institute of Biotechnology, University of Helsinki, Helsinki, Finland; 5 Encepharm, Göttingen, Germany; 6 Department of Nuclear Medicine, University Medical Center, Georg-August-University Göttingen, Göttingen, Germany; 7 Department of Molecular Embryology, Institute for Anatomy and Cell Biology, Albert-Ludwigs-University Freiburg, Freiburg, Germany; 8 Department of Cognitive Neurology, University Medical Center, Georg-August-University Göttingen, Göttingen, Germany; Florey Institute of Neuroscience and Mental Health, The University of Melbourne, AUSTRALIA

## Abstract

Cerebral dopamine neurotrophic factor (CDNF) belongs to a newly discovered family of evolutionarily conserved neurotrophic factors. We demonstrate for the first time a therapeutic effect of CDNF in a unilateral 6-hydroxydopamine (6-OHDA) lesion model of Parkinson’s disease in marmoset monkeys. Furthermore, we tested the impact of high chronic doses of human recombinant CDNF on unlesioned monkeys and analyzed the amino acid sequence of marmoset CDNF. The severity of 6-OHDA lesions and treatment effects were monitored *in vivo* using ^123^I-FP-CIT (DaTSCAN) SPECT. Quantitative analysis of ^123^I-FP-CIT SPECT showed a significant increase of dopamine transporter binding activity in lesioned animals treated with CDNF. Glial cell line-derived neurotrophic factor (GDNF), a well-characterized and potent neurotrophic factor for dopamine neurons, served as a control in a parallel comparison with CDNF. By contrast with CDNF, only single animals responded to the treatment with GDNF, but no statistical difference was observed in the GDNF group. However, increased numbers of tyrosine hydroxylase immunoreactive neurons, observed within the lesioned caudate nucleus of GDNF-treated animals, indicate a strong bioactive potential of GDNF.

## Introduction

Neurotrophic factors are considered potent candidates for the disease modifying treatment of neurodegenerative disorders such as Parkinson’s disease (PD). The newly discovered cerebral dopamine neurotrophic factor (CDNF) gained attention because it restored function and promoted survival of midbrain dopaminergic (DA) neurons in 6-hydroxydopamine (6-OHDA) and 1-methyl-4-phenyl-1,2,3,6-tetrahydropyridine (MPTP) models of PD in rodents when delivered intracranially either as a recombinant protein or with adeno-associated viral serotype 2 (AAV2) vector as treatment regimens in rodent models of PD [1–6].

CDNF and related protein mesencephalic astrocyte-derived neurotrophic factor (MANF) represent an evolutionarily conserved family of neurotrophic factors with a unique structure and mode of action [1], [7–10]. The CDNF amino acid sequence is found in vertebrates [1], whereas invertebrates have a single homolog more related to mammalian MANF than to CDNF [11]. The three dimensional structure of MANF/CDNF proteins consist of two domains [7, 9] and eight cysteine residues determining the domain folding are conserved across species. Although MANF/CDNF proteins can be secreted [1, 12] they also function in the endoplasmic reticulum (ER) and the protective role for MANF against ER stress has been demonstrated [13–15].

Glial cell line-derived neurotrophic factor (GDNF) has been considered the most promising neurotrophic factor, showing positive effects in several rodent and nonhuman primate models of PD [16–24], but by contrast with CDNF, it had very modest effects in a severe rat 6-OHDA model of PD [3]. Interestingly, GDNF had no neuroprotective effects in the severe alpha-synuclein (α-syn) model of PD [25–26], because α-syn downregulates transcription factor Nurr1 and consequently GDNF signaling receptor RET, disrupting GDNF signalling in DA neurons [27]. In PD patients, gene therapy using neurturin (NRTN), a member of the GDNF family ligands showed a modest clinical benefit compared to placebo controlled trials [28].

Controversial results from clinical trials with GDNF protein [29–32] and NRTN gene therapy have highlighted the importance of effective and reliable administration techniques (discussed by Sherer and colleagues [33]) [34–37]. Moreover, GDNF and NRTN are basic proteins that bind with high affinity to heparin sulfate proteoglycans in the extracellular matrix [38], which restricts their diffusion in the brain tissue [39]. We have recently shown that CDNF and MANF are neutral proteins that diffuse significantly better than GDNF in rodent brains [2–3]. These results together with CDNF efficiency to protect and repair DA neurons in rodent midbrain warrant testing for its efficacy in nonhuman primate models of PD.

Nonhuman primates, e.g. common marmosets (*Callithrix jacchus*), share similarities with humans regarding complex brain function and drug safety. Potent compounds such as neurotrophic factors can cause undesired effects as reported for nerve growth factor (NGF) and GDNF when applied in high doses into the brain [40–42]. The present tolerability study aimed to define experimental parameters for a safe intracerebral application of CDNF protein in the future.

The impact of lesion severity on brain integrity and repair processes should be considered when studying neuroregeneration [43–48]. Of note, neuroprotective approaches were generally more successful than neurorestorative approaches [1, 22], and this fact may be attributed to the chronic neurodegeneration of DA neurons in the 6-OHDA model, especially after nigral and nigrostriatal bundle lesions [46, 49]. With regard to translational aspects, severe 6-OHDA lesions chiefly mimic the endpoint of Parkinson’s disease, where the majority of DA neurons are lost and are therefore of limited use for therapeutic studies [46]. In our concept of a successful neurorestorative treatment approach, the endogenous regenerative capacity of the brain needs to be conserved. Consequently, establishing a 6-OHDA protocol for mild lesions in marmosets was required.

In the presented study we induced mild 6-OHDA lesions into the caudate nucleus of common marmosets as a preclinical model for neurodegeneration. The aim of the study was to test the therapeutic efficacy of CDNF and to compare it with GDNF in a neurorestorative treatment approach. Striatal DA neurodegeneration was monitored *in vivo* by following dopamine transporter integrity using ^123^I-FP-CIT SPECT. To provide an additional aspect on biosafety, we assessed the tolerability of high CDNF doses. Moreover, we determined the marmoset CDNF amino acid sequence and compared it within primates.

## Materials and Methods

### Ethics Statement

The animals were obtained from the breeding colony at the German Primate Center (Göttingen, Germany) and pair-housed in a temperature- (25 ± 1°C) and humidity-controlled (65 ± 5%) facility under a 12 h day/night cycle. Each cage (80 x 150 x 66 cm, Ebeco GmbH, Castrop-Rauxel, Germany) was furnished with wooden branches and shelves and contained a wooden sleeping box (24 cm x 21 cm x 18 cm). The animals were fed *ad libitum* with a pelleted marmoset diet (Ssniff Spezialdiäten, Soest, Germany). In addition, a mash which contains in the dry matter 21% crude protein, 14% fat, 10% crude fiber, 41% starch and saccharides, 0.95% and Ca, 0.67% was served in the morning. Per animal 20 g of the mash with a dry matter content of 38% was offered. In the afternoon each animal received 30 g clean-cut fruit or vegetables mixed with noodles or rice. Water was always available. Behavior of the animals was observed on a daily basis and their weight was measured every week. All animal experimentation was carried out in accordance with the European Council Directive of September 2010 (2010/63/EU), and was approved by the Lower Saxony Federal State Office for Consumer Protection and Food Safety, Germany (permit numbers: 04-076/09, 04-10/0161 and 04-11/0394).

### Animals

Four adult male (3–4 years old) and two female (3 and 6 years old) common marmoset monkeys (*Callithrix jacchus*) (337-485g) were used for the chronic CDNF infusion study. Eight adult (3–7 years old) male and one female (9 years old) animal (375-550g) were used for the treatment study. One adult female (2 years old) animal was used for CDNF sequence analysis.

### Chronic intracerebral delivery of CDNF

**Fig 1:**
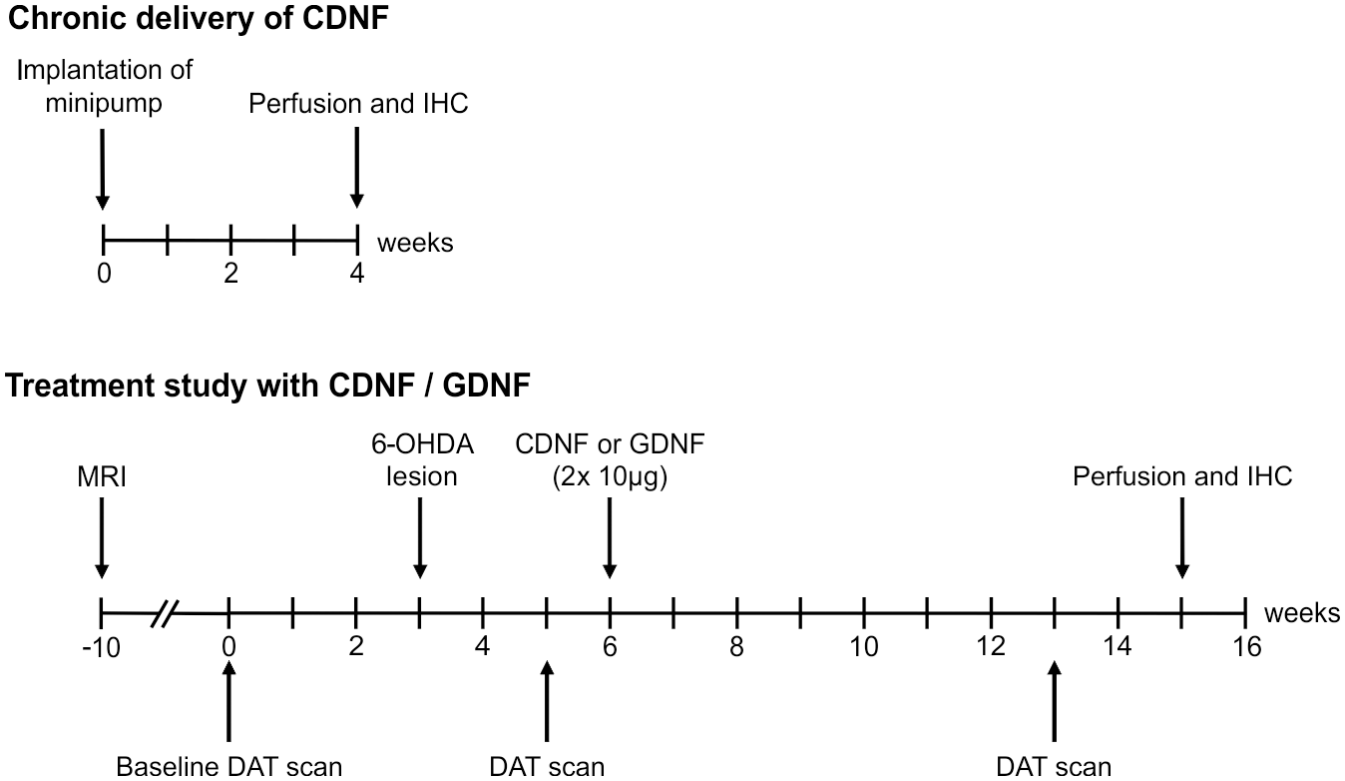
Time course of experiments. In the tolerability approach (upper scheme) high concentrations of CDNF (10 or 15μg per day) were intrastriatally delivered for 28 days via osmotic minipumps followed by subsequent histopathological assessment. Efficacy of CDNF and GDNF was tested in the 6-OHDA model (lower scheme). Treatment effects were evaluated *in vivo* by SPECT imaging and post mortem IHC.

CDNF protein was chronically delivered into the right caudate nucleus via an implanted cannula connected to an osmotic pump. Surgery was carried out under inhalation anaesthesia [60:40 N_2_O:O_2_; isoflurane (0.5–1.5%)] and diazepam (Ratiopharm, Germany; 0.05 mg per animal) anaesthesia and aseptic conditions. A Teflon infusion cannula (PlasticsOne, Roanoke, VA, USA) was inserted stereotactically into the right caudate nucleus (AP+8.0 mm; L+3.0 mm, V+13.0 mm) according to the atlas by Stephan and colleagues [50]. After the injection cannula was inserted into the brain, it was fixed to the skull with dental cement. An Alzet osmotic minipump (model 2004, 0.25μl/h; Charles River, Sulzfeld, Germany) was implanted subcutaneously in the retroscapular area and connected to the infusion cannula via the catheter tube to deliver either CDNF or 0.1 M phosphate buffered saline (PBS), pH 7.2 for a period of 4 weeks. Human recombinant CDNF was expressed and purified as previously described [1] with additional removal of tag sequences by thrombin cleavage. The pumps were filled with CDNF dissolved in PBS and 0.1% bovine serum albumin (BSA). As a control, pumps were filled with PBS and 0.1% BSA alone. Four animals received 15μg CDNF per day (CDNF concentration 2.5 μg/μl, 6 μl/24 h), and one animal received 10μg CDNF per day (CDNF concentration 1.7 μg/μl, 6 μl/24 h), chronically delivered for 4 weeks (Fig 1). Thus, one group of the animals received a total of 420 μg of CDNF and the other group 280 μg. A single animal received PBS as a control. Postoperatively, the animals were treated with meloxicam (0.2 mg/kg i.m., Metacam, Boehringer Ingelheim, Germany) for 3 days and enrofloxacin (5 mg/kg i.m., Baytril, Bayer, Germany) for 5 days.

### Mild unilateral 6-hydroxydopamine lesion and intracerebral injection of neurotrophic factors

Marmosets were given unilateral 6-OHDA injections into the caudate nucleus. Anesthesia was induced and maintained as described above. 6-OHDA (1.66 mg/ml free base weight 6-OHDA hydrobromide [Sigma-Aldrich, Steinheim, Germany] dissolved in 0.01% ascorbate-saline) was injected stereotactically into six sites (5.81 μg/3.5 μl each injection) within the caudate nucleus on one side of the brain. Coordinates were determined according to the atlas by Stephan and colleagues [50]: first site: AP+7.5 mm, L+2.5 mm, V+12.5 mm; second site: AP+7.5 mm, L+3.5 mm, V+13.5 mm; third site: AP+9.0 mm, L+2.5 mm, V+12.0 mm; fourth site: AP+9.0 mm; L+3.5 mm, V+13.0 mm; fifth site: AP+10.5 mm, L+2.25 mm, V+12.0 mm; and sixth site: AP+10.5 mm, ML+3.25 mm, V+13.0 mm). In all cases, 6-OHDA solution was freshly prepared and stored on ice just before use. Each injection of 6-OHDA was made at a rate of 0.5 μl/min using a glass capillary (Nano injector 2000, WPI, Berlin, Germany), which was left in place for a further 4 min after each injection had been made. Following surgery, the monkeys were provided analgesia (as above) and kept in a warm incubator until they were well enough to be returned to their home cages. Postoperatively, the animals were treated with analgesics and antibiotics as described above. Three weeks after 6-OHDA lesion induction 20 μg of either CDNF (n = 4 animals) or GDNF (n = 4 animals) (PeproTech, London, UK) (two injections of 10 μg/3 μl of one of neurotrophic factor, dissolved in PBS) was intracerebrally injected (as described above for 6-OHDA injections) at two sites at the center of the lesion using the following coordinates: first site: AP+8.25 mm, L+3.0 mm, V+13.0 mm; second site: AP+9.75 mm, L+3.0 mm, V+12.5 mm. In a previous pilot trial we tested different experimental settings and SPECT acquisition time points. Based on individual SPECT scans we assume that recovery of DAT activity to normal levels is unlikely to occur in PBS treated animals. Therefore, we omitted this group in the treatment study, except of a single PBS-treated animal (S4 Fig), and focused on the comparison with the potent compound GDNF.

### Physical and neurological examination

Clinical examinations included general condition [body weight, food consumption and excretion (faeces), exsiccosis, skin condition, attention] and neurological status (sensory limb reflex, handedness, movement activity pupillary reflex, eye tracking, orbicularis oculi reflex, gait, and tremor). In addition, animals were observed in their home cages to assess their behavior (attention, movement activity, and gait). Animals were examined in average twice weekly and at least for three days in a row after each surgical intervention. For the treatment study, examinations were discontinued for one week after every imaging session (SPECT, MRI). In the treatment study body weights were not included for evaluation due to possible impact of repeated anaesthetic procedures (animals were fasted prior to anaesthesia).

### SPECT imaging

SPECT imaging was performed as previously described [49]. Briefly, a three-headed gamma camera (Prism 3000XP, Picker International, Cleveland, OH, USA) was upgraded with a dedicated small-animal imaging module (HiSPECT, Scivis, Göttingen, Germany). Each collimator head was equipped with a pyramidal collimator and multi-pinhole aperture plate (six pinholes). The inner diameter of each pinhole was 2.5 mm, which yielded a tomographic resolution of 2.1 mm and an average of axis sensitivity of 960 cps/MBq. The field of view (FoV) was 143 cm^3^. Images were acquired at 256 x 256 projection matrices with a pixel size of 1.78 mm over 10 gantry steps (30 projection angles) in a step-and-shoot mode. Up to four energy windows were acquired. An acquisition time of 300 s per gantry step and total scan time of 50.2 min was chosen. The photo peak energy was set to 159 keV with a window of ±10%. Multi-energy window scatter correction was applied. The anesthetized animals were placed in a custom-made holder in a prone position and the head was fixed with ear bars. Image acquisition was initiated after reaching equilibrium state 2 h after injection of 50–60 MBq ^123^I-FP-CIT (0.6–0.8 ml) (Datscan, Amersham Buchler, Braunschweig, Germany). SPECT reconstruction was performed using a dedicated iterative ordered subset expectation maximization (OSEM) algorithm consisting of three iterations and four subsets per iteration (HiSPECT). The reconstructed voxel size was 0.6 mm^3^. For quantification, brain activity was determined with HiSPECT software. Rectangular-shaped regions of interest (ROIs) of 991.4 mm^3^ volume were chosen manually for the left (STl) and right striatum (STr) and cerebellum (CB, 370.3 mm^3^) using corresponding magnetic resonance imaging (MRI, see below). Specific binding in the ST was calculated by subtracting the mean activity in the CB from the mean activity in the ST and dividing the result by the mean activity in the CB, i.e. (ST-CB)/CB. The binding ratio of the contralateral site served as an internal control and was set to 100%. For longitudinal monitoring of DAT activity a total of three scans per animals were performed (Fig 1).

### Magnetic resonance imaging

For providing a reference of local brain anatomy, MRI was performed on a 3T clinical MR system (Magnetom TIM TRIO, Siemens Healthcare, Erlangen, Germany) [51]. Marmosets were scanned in supine position in the wrist coil under general anesthesia with diazepam (0.3 mg/kg i.m.), alfaxolon (10 mg/kg i.m, Alfaxan, Vetoquinol, UK), and glycopyrronium bromide (0.01 mg per animal i.m., Robinul, Riemser, Germany). The total measurement time for structural MRI at 0.4 mm isotropic resolution was about 10 min, thus eliminating the need to intubate the animal.

The three-dimensional (3D) imaging volume covered the head and upper chest with the read-out direction along the body axis. The FoV in the right-left and ventral-dorsal directions was 51.2 mm. T1-weighted (T1-w) contrast was achieved by magnetization-prepared rapid acquisition of gradient echoes (MP-RAGE) using parameters recommended for humans (inversion time TI = 0.9 s, flip angle α = 9°, bandwidth BW = 200 Hz/pixel, echo time TE = 4.06 ms, repetition time TR = 2.25 s) [52]. The matrix of 320 x 128 x 128 was acquired in 4:18 min. T2-w contrast was achieved by a 3D turbo spin echo (TSE) sequence with variable refocusing angle to preclude unexpected brain lesions. The matrix of 256 x 128 x 128 was acquired in 5:26 min.

### Post-processing of imaging data

The 2D digital imaging and communications in medicine (DICOM) MRI images in axial-to-coronal orientation were converted to 3D neuroimaging informatics technology initiative (NIFTI) volumes, changing from the radiological right-left convention to the left-right convention of the SPECT images. The T1-w volume was aligned to a custom-made T1-w template of the marmoset head and interpolated to 0.25 mm resolution to achieve consistent angulation of the horizontal plane. This was defined by the intercommissural line, as featured in the stereotaxic atlas of the National Institute of Neuroscience, Japan [53]. This T1-w volume then served as an individual anatomical reference to align the T2-w and SPECT volumes, using the FMRIB software library (FSL 4.1, Centre for Functional Magnetic Resonance Imaging of the Brain, University of Oxford, UK, www.fmrib.ox.ac.uk/fsl). For spatial alignment, the FSL linear registration tool (FLIRT), was used to determine the 3D rigid body transform (six parameters) by a mutual information criterion.

### Cloning of marmoset CDNF cDNA and sequence analysis

Total RNA from the hippocampus was isolated using Tri-reagent (Thermo Fisher Scientific, Helsinki, Finland) following the manufacturer’s instructions. First-strand cDNAs were synthesized with Revertaid Premium reverse transcriptase (Thermo Fischer Scientific) according to the manufacturer's instructions, using oligo(dT) primed total RNA (1 μg) as a template. The following PCR primers containing either *Bam*HI or *Hind*III restriction sites were used: cj-CDNF_*Hind*III_F (5'- ctctaagcttATGTGGTGCGCGAGCCCAGC -3') and cj-CDNF_*Bam*HI_stop_R (5'- ataaggatccTCAGAGCTCTGCTTTGGGGTGTG -3') or cj-CDNF_*Bam*HI_nostop_R (5'- ataaggatccGAGCTCTGCTTTGGGGTGTG -3'). PCR reactions were performed using the Phusion Hot Start II High-Fidelity DNA polymerase (Thermo Fisher Scientific). PCR products were restricted with *Bam*HI and *Hind*III and were cloned into *Bam*HI and *Hind*III sites in pCR3.1 vector (Invitrogen, Carlsbad, USA) and sequenced.

Alignment of amino acid sequences was performed with CLUSTAL O (version 1.2.0) software. Signal peptide cleavage sites were predicted using the SignalP 4.1 server (http://www.cbs.dtu.dk/services/SignalP/). Amino acid identity (%) of CDNF proteins was calculated by EMBOSS Needle program http://www.ebi.ac.uk/Tools/psa/emboss_needle/.

### Histology

At the end of the experiments, animals were sacrificed for further histological evaluation. Animals underwent deep anaesthesia using a cocktail of ketamine, xylazine, and atropine. Subsequently, animals were transcardially perfused with 0.9% NaCl and 4% paraformaldehyde in PBS, pH 7.2. After storage of the heads in the fixative for 24 h at 4°C the brains were carefully removed, post-fixed in 4% paraformaldehyde in phosphate buffered saline and stored at 4°C until analysis. For the chronic CDNF infusion experiments, 3-mm-thick coronal brain slices were embedded in paraffin and cut into 3-μm-thick sections. Hematoxylin and eosin (HE) staining was performed for standard microscopic pathology evaluation of relevant brain structures (four slices of forebrain including midbrain, and two slices of hindbrain including cerebellum). As no abnormalities were observed in remote brain areas, we limited immunohistochemical evaluation on the coronal plane in the vicinity of the infusion site. For immunohistochemistry tissue sections were treated with citrate buffer (10 mM sodium citrate, pH 6). Immunohistochemistry was performed to assess inflammation using antibodies against CD3^+^ T-cells (1:50, A452, Dako, Hamburg, Germany), CD 20^+^ B-cells (1:200, M755, Dako) and MAC^+^ macrophages/ activated microglia (1:300, M0747, Dako). Discovery universal secondary antibody (760–4205, Discovery universal secondary antibody, Roche, Basel, Switzerland) was used. Antibodies were visualized using DAB detection kit (760–124, DABMap, Roche, Basel, Switzerland). Slides were counterstained using Mayer’s hemalaun solution. Control sections were incubated in absence of primary antibody. Lymph nodes of healthy marmosets served as positive control for inflammatory markers. The degree of inflammation within the injected brain hemisphere was assessed on representative on HE-stained and CD3-stained coronal sections using a semiquantitative score (- no, + mild, ++ moderate, +++ severe). Regions of interest were the vicinity of cannula track (a region of approx. 50 μm around the cannula track, referred as “infiltrates in the vicinity of cannula track”) and a broad area (including striatum, corpus callosum and cerebral cortex, referred as “widespread perivascular infiltrates”).

For the treatment study, post-fixed brains were stored in 30% sucrose, frozen and cut into 40-μm-thick sections. Antibodies against CDNF (1:1000, #4343, ProSci, Poway, CA, USA), tyrosine hydroxylase (1:500, Biotrend, Cologne, Germany) and dopamine transporters (1:1000, D6944, Sigma-Aldrich) were used overnight. Appropriate secondary antibodies were used (1:200, Vector Laboratories, Axxora Deutschland GmbH, Lörrach, Germany). Sections were treated with avidin-biotin-peroxidase complex (PK-6100, Vectastain Elite ABC, Vector Laboratories). DAB was used as the chromogen (Vector Laboratories). Control sections were incubated in absence of primary antibody. Digital images of immunostained tissue sections were acquired using an Axiophot II microscope (Zeiss, Germany). Distribution of CDNF after chronic delivery was assessed on CDNF-ir representative coronal sections using a semiquantitative score (- no signal, + low, ++ moderate, +++ high).

Numbers of TH-immunoreactive (TH-ir) neurons in the caudate nucleus were assessed on coronal TH-stained sections. 10–12 representative sections at interval of 30 μm were used to cover the rostro-caudal extension of the striatum of each animal. Counting of TH-immunoreactive cells was performed with an Axioplan II light microscope (Zeiss) using a counting grid on 10-fold magnification placed on all caudate areas of the section. Only cells positive for TH that possessed a clear and vital neuronal morphology were included for counting. The number of positive cells per cm^2^ was reported. Numbers of TH-ir neurons in the *substantia nigra pars compacta* (SNc) were assessed on coronal TH-stained 30μm thick sections. Every 8^th^ section was used to cover the rostral-caudal extension of the SNc. Counting of TH-ir neurons was performed as described above.

### Statistical analysis

Data are presented as mean ± standard deviation (SD). Data were analyzed using GraphPad Prism (version 5.00 for Windows, GraphPad Software, San Diego, CA, USA) and SigmaPlot 11 (Systat Software Inc., San Jose, CA, USA). Statistical analyses of the SPECT study included repeated measures analysis of variance (ANOVA) followed by post hoc Holm-Sidak test and Bonferroni-Holm correction for adjusted p values. Unpaired Student’s t-tests were performed for quantitative histological analysis. Differences were considered statistically different if p < 0.05. Data analysis was performed in a blind fashion.

## Results

### Histopathological changes after high chronic CDNF infusion

The tolerability of CDNF was tested starting with low concentrations of the growth factor protein. Because no pathological effects were detected after a single intracerebral injection of 30 μg CDNF in previous experiments (S1 Fig), higher doses of CDNF were delivered chronically for 4 weeks using osmotic pumps. Histopathologically, no abnormalities were detected in the marmoset receiving 10 μg CDNF per day (with a total amount of 280 μg CDNF over a 4-week period of delivery) and the vehicle control. Therefore, we focussed on higher doses in the actual experiments.

**Fig 2:**
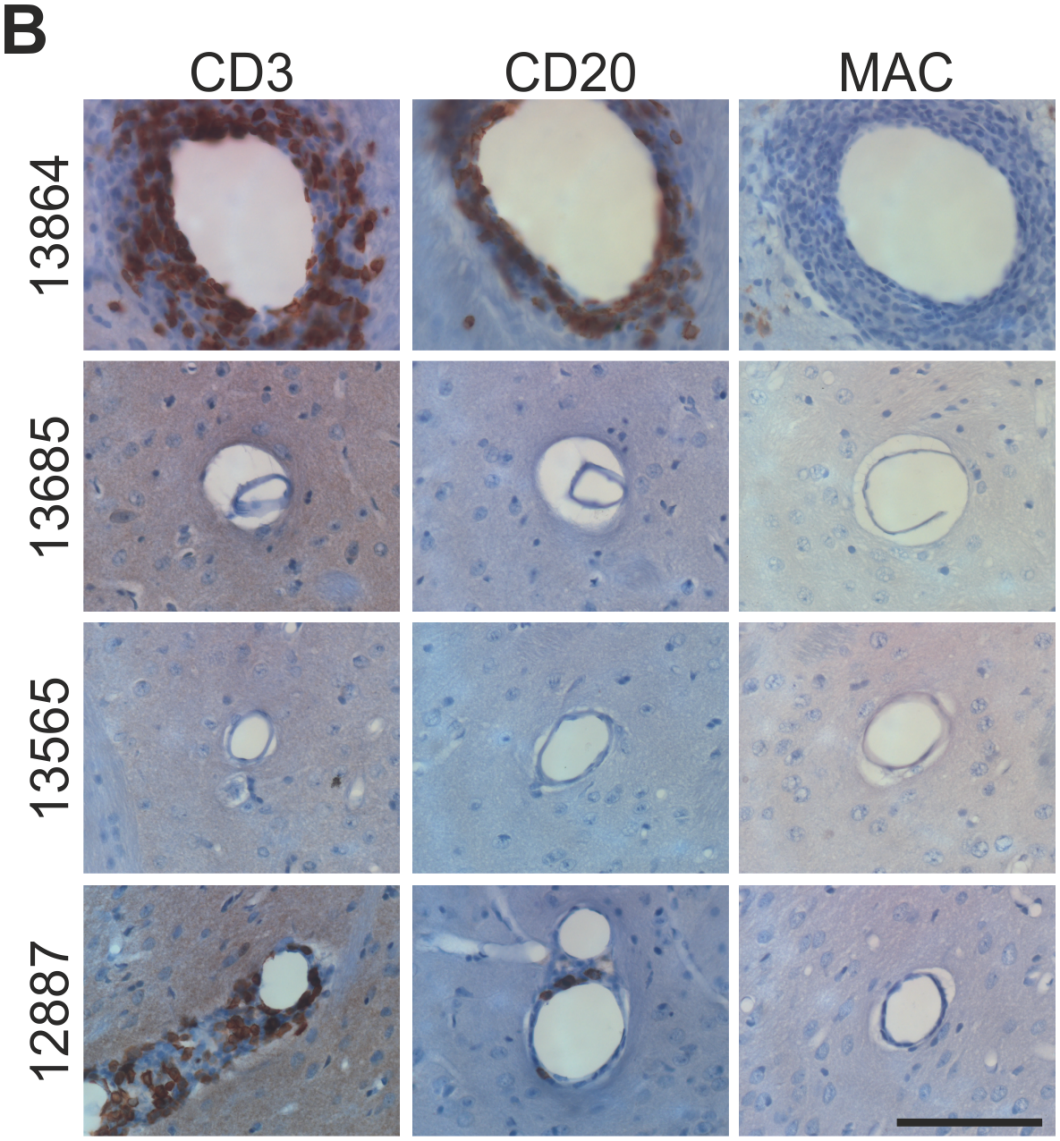
Histopathological assessment of the caudate nucleus of chronically CDNF-infused animals (CDNF: 15μg/day for 28 days; totally 420 μg). Perivascular infiltrates positive for CD3 and CD20 were observed in two animals (animals # 13864 and # 12887) out of four analysed. MAC-positive macrophages/activated microglia were absent. Scale bar = 100μm.

Four marmosets received a chronic CDNF infusion of 15 μg/day for 28 days (420 μg CDNF in total). In two animals (Fig 2, animals # 13685 and # 13565), no obvious histopathological abnormalities were observed. In a single animal histopathological analysis revealed a strong parenchymal and perivascular reaction composed of CD3^+^ and CD20^+^ infiltrates in the absence of macrophages or activated microglia (Fig 2, animal # 13864). A fourth animal (animal # 12887) showed a local, moderate inflammation restricted to perivascular areas in the vicinity of cannula track. These results indicate potential immunogenicity of CDNF in single animals when administered in high doses. However, semiquantitative analysis of CDNF-ir tissue distribution, revealed that CDNF-ir was completely absent in a single high-dose CDNF animal (S2 Fig, Table 1, animal # 13685) and this might explain the absence of histopathology in this particular animal. Moreover, the distribution pattern of infiltrates observed in animal # 12887 is partly matching with CDNF-ir distribution. However, we cannot rule out the possibility that injected CDNF had become contaminated and caused the observed histopathology in two of four animals. Besides the mentioned histopathology, the general condition of the animals was not affected. Body weights remained stable (S3 Fig) and the animals showed no abnormal behavior. The CDNF dose used in the neurorestorative treatment approach was about 15 times less than the maximum tolerated dose.

**Table 1:** Tolerability of chronic CDNF delivery: concentrations of CDNF in comparison to the degree of histopathology and CDNF-ir tissue distribution. Histopathological scoring was based on HE-staining and CD3 immunohistochemistry (- no, + mild, ++ moderate, +++ severe). CDNF-ir distribution (See also S2 Fig) was semi quantitatively assessed on coronal sections (- no signal, + low, ++ moderate, +++ high).

		Histopathology		
Animal ID	Treatment	Infiltrates in the vicinity of cannula track	Widespread perivascular infiltrates	CDNF-ir distribution
13864	15μg CDNF	+++	+++	+++
13685	15μg CDNF	+	-	-
13565	15μg CDNF	-	-	+++
12887	15μg CDNF	++	+	++
13601	10μg CDNF	-	-	+
13639	PBS	-	-	-

### CDNF increases striatal DAT activity after mild 6-OHDA lesions

**Fig 3:**
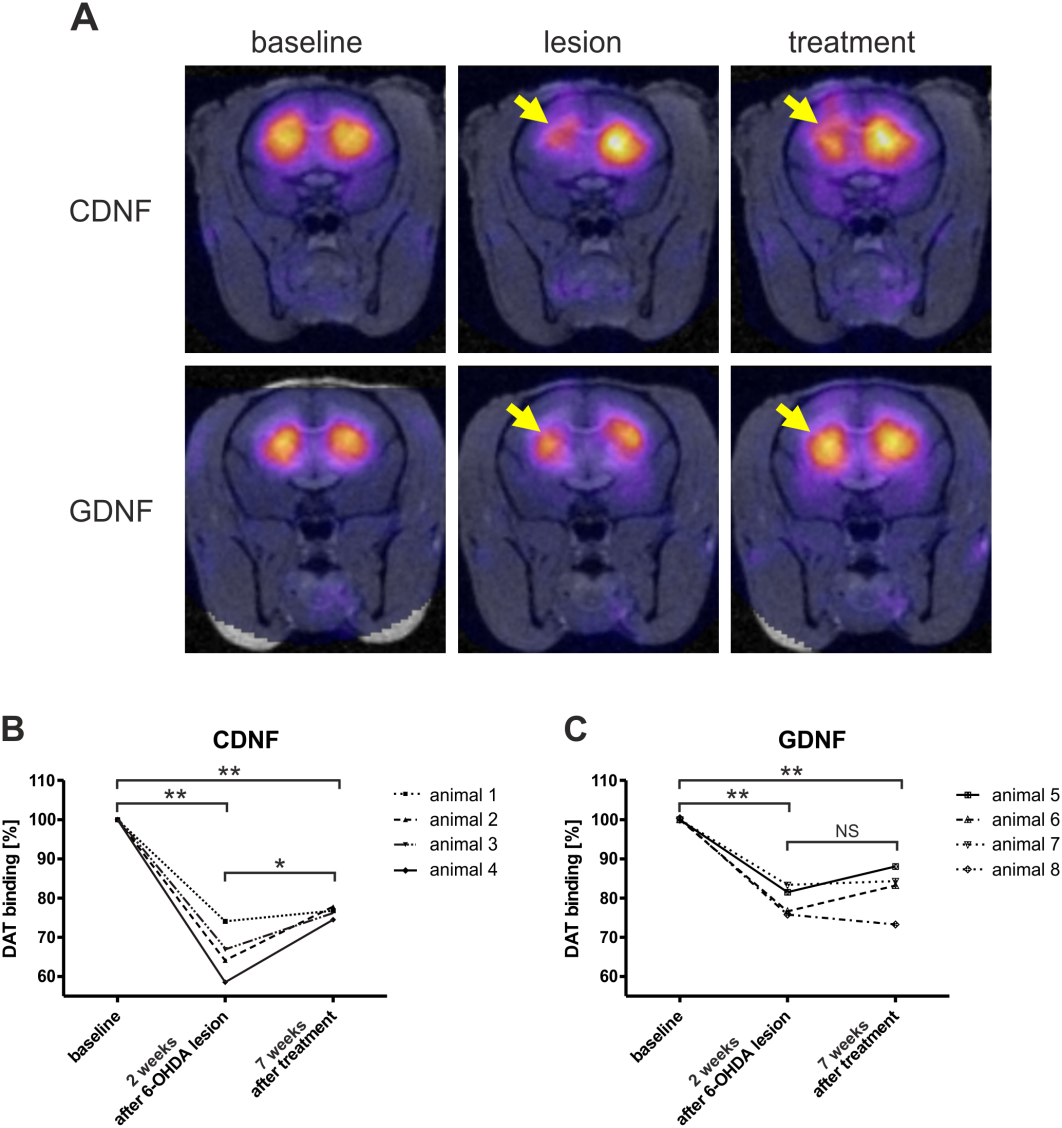
(A) Representative ^123^I-FP-CIT SPECT images of CDNF (upper row) and GDNF treated (lower row) marmosets. Reduced DAT-binding was observed after mild 6-OHDA lesions when targeted to the caudate nucleus (middle row, indicated by arrows). Treatment via intracerebral injection of either 20 μg of CDNF (upper row) or 20 μg of GDNF (lower row), lead to obvious recovery on the level of DAT integrity (indicated by arrows). (B, C) Treatment effects were evaluated by quantitative analysis of ^123^I-FP-CIT SPECT. Animals showed a significant increase of DAT-binding (*p = 0.023, [F_2,11_ = 33.32, p < 0.001]) when treated with CDNF (B). In the GDNF group single animals responded to the treatment with GDNF (C, animals #5 and #6). However, no significant recovery was observed in the GDNF-treated group. *p < 0.05, **p < 0.01, NS = not significant.

To maintain the regenerative capacity of the brain a mild 6-OHDA lesion protocol was established. Injections of 6-OHDA were targeted to the caudate nucleus leading to a notable but mild reduction of dopamine transporter activity (Fig 3). 6-OHDA lesions did not lead to overt behavioral impairment or loss of substantia nigra neurons (S1 Table). In some cases, transient and inconsistent tremor was observed within the first 2 weeks after lesioning.

Quantitative SPECT analysis of the affected brain area confirmed a moderate reduction of striatal DAT binding with values of 65.91 ± 6.47% in the CDNF group and 79.31 ± 3.69% in the GDNF group, compared with their corresponding contralateral untreated sides (Fig 3B and 3C). Three weeks after inducing lesions, the animals received intracerebral injections of the neurotrophic factors. In CDNF-treated animals, we observed a significant (*p = 0.023, [F_2,11_ = 33.32, p < 0.001]) recovery of striatal DAT binding of approximately 10% up to 76.32 ± 1.38% 8 weeks after treatment. By contrast, in the GDNF-treated group, DAT binding only slightly increased, on average to 82.20 ± 6.31%. Two animals in the GDNF-treated group responded to the treatment (approximately 7% recovery). Two other animals did not show any obvious change in DAT binding after GDNF treatment. In summary, no statistical differences were observed in the GDNF group. As a control a single animal was treated with PBS after 6-OHDA lesion paradigm. No spontaneous recovery was observed in DAT imaging (see S4 Fig).

**Fig 4:**
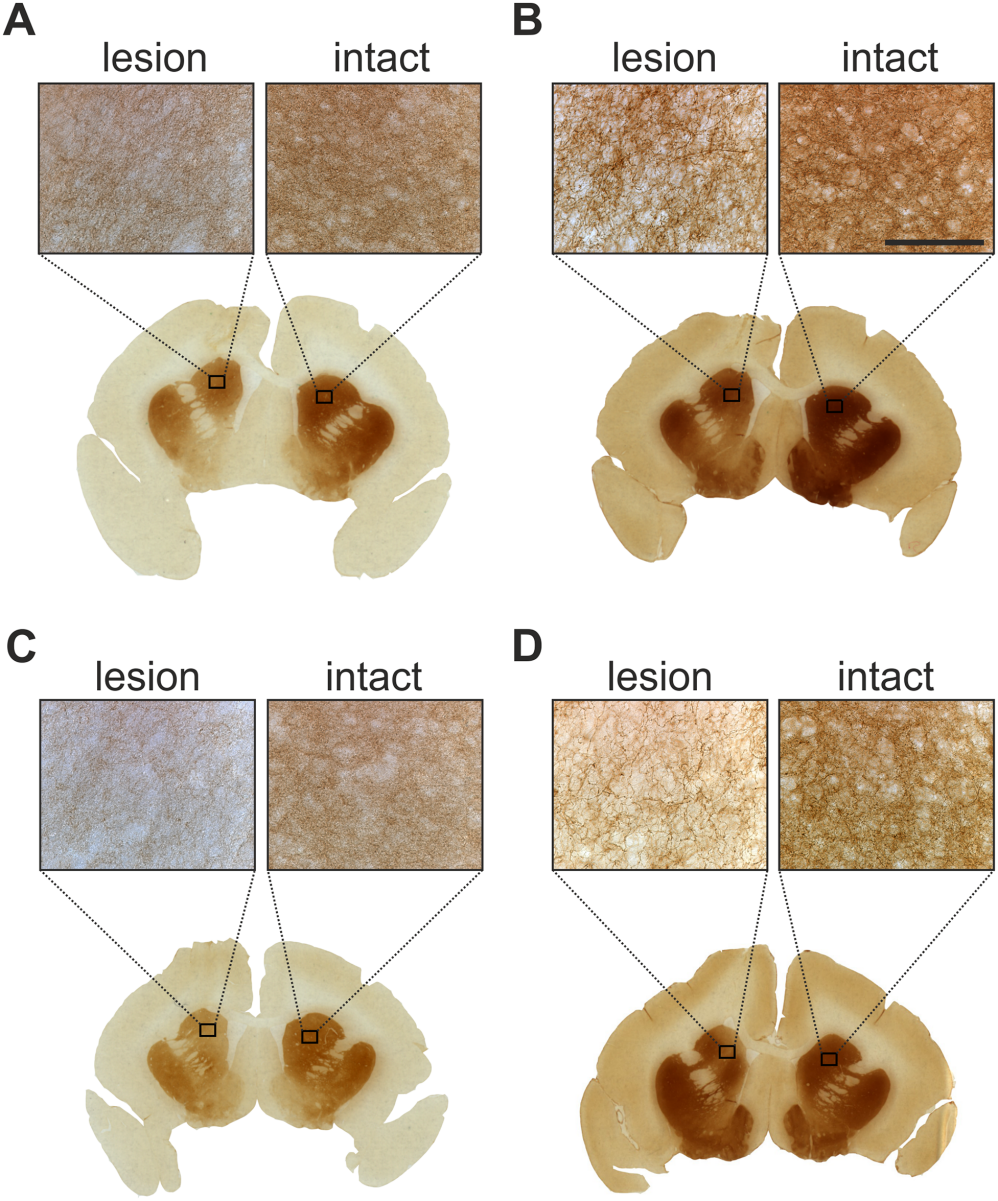
Immunohistochemistry of the caudate nucleus was performed at the end of experiment. Sections were stained for DAT (A / C) and TH (B / D). The affected brain areas showed moderate dopaminergic integrity after treatment with CDNF (A / B) and GDNF (C / D). Scale bar: 100μm.

**Fig 5:**
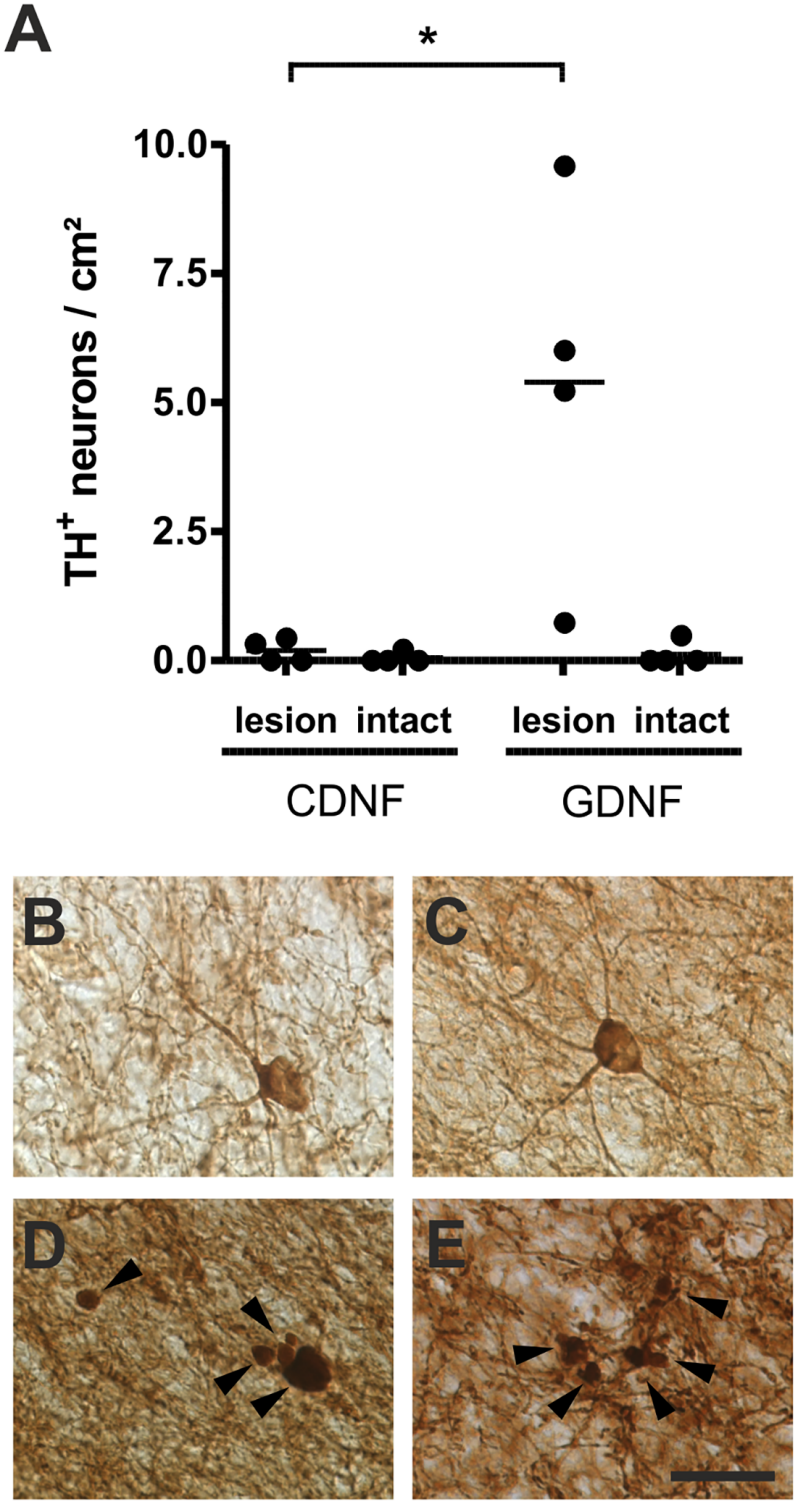
Structural effects of GDNF treatment on TH-immunoreactivity was observed within the lesioned caudate nucleus (B—E). TH-positive neurons showed a typical branching pattern (B/C). The number of TH-positive neurons was significantly increased in the GDNF treated lesioned caudate nucleus (A) compared to CDNF treated animals (p < 0.05). Moreover, a high fraction of small TH-positive structures (D) and TH-positive varicosities (E) were observed within the GDNF-treated lesioned caudate. Mean. *p < 0.05. Scale bar, 25μm.

At the end of the experiment, a histological examination was performed (Fig 4). The topography and intensity of TH and DAT immunoreactivity of the lesioned caudate nucleus corresponded to the picture observed in SPECT. However, detailed analysis revealed structural changes on a microscopic level within the lesioned caudate nucleus of GDNF-treated animals. High numbers of TH-positive cells were observed within the lesioned area, and many of them could be clearly assigned to vital neurons because they possessed a typical branching pattern (Fig 5B and 5C). Furthermore, a high fraction of small TH-positive structures (Fig 5D) and TH-positive varicosities (Fig 5E) were observed within the GDNF-treated lesioned caudate. Interestingly, no structural changes were observed in TH-stained sections of CDNF treated animals. Moreover, no obvious changes were observed in DAT immunoreactivity in any of the experimental groups.

Quantification revealed increased numbers of TH-positive neurons in the lesioned GDNF-treated caudate nucleus (Fig 5A, 5.39 ± 3.64 cells/cm^2^). This effect was significant (*p < 0.05, unpaired Student’s t-test) when compared with the lesioned CDNF-treated caudate nucleus (0.19 ± 0.23 cells/cm^2^). As expected, TH-positive neuronal counts were negligible in the contralateral site of marmosets from the GDNF- (0.12 ± 0.24 cells/cm^2^) and CDNF-treated groups (0.05 ± 0.11 cells/cm^2^). Quantification of TH-ir in midbrain showed no statistical difference between lesioned and contralateral SNc and treatment groups, respectively (S1 Table).

### Amino acid sequence of marmoset CDNF

**Fig 6:**
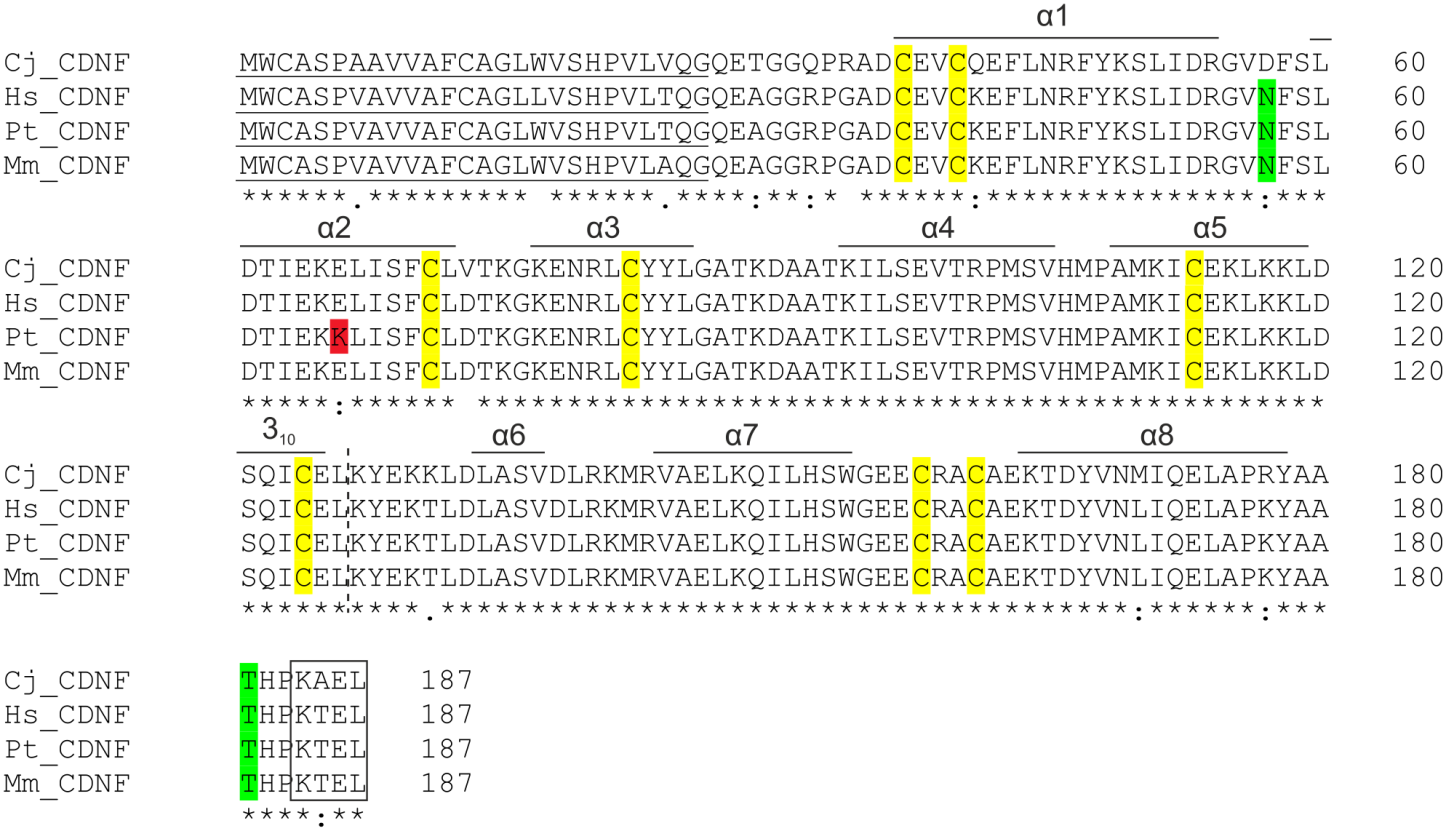
Alignment of marmoset (Cj), human (Hs), chimpanzee (Pt), and macaque (Mm) CDNF amino acid sequences by CLUSTAL O (version 1.2.0) software. Identical amino acids are marked with an asterisk, physicochemically highly similar with a colon, and similar with a dot. Predicted signal sequences (SignalP 4.1 Server http://www.cbs.dtu.dk/services/SignalP/) of marmoset, chimpanzee and macaque CDNF (residues 1–26; underlined) are equal in length to the verified signal peptide of human CDNF [1]. Eight conserved cysteine residues with identical spacing of the mature CDNF are indicated in yellow. N-linked glycosylation site of human CDNF at position 57N [13] and O-linked glycosylation site at position 181T [52] is indicated in green. The C-terminal ER-retention motif KAEL/KTEL is boxed. Alpha-helical regions of CDNF are indicated above the sequences according to [7] (helices α1-α5 and turn of 3_10_ helix) and [9] (helices α6-α8). The division between N-terminal saposin-like domain and C-terminal SAP-domain is indicated by a vertical dashed line.

To verify the *Callithrix jacchus* CDNF sequence we cloned a corresponding cDNA from the hippocampal tissue of a female marmoset. The obtained nucleotide sequence was identical to the coding sequence of GenBank GAMQ01005170 mRNA (translation JAB36681.1). Alignment of CDNF amino acid sequences from marmoset, human [1], chimpanzee (*Pan troglodytes*; GenBank prediction XP_507666.2) and rhesus macaque (*Macaca mulatta*; GenBank prediction NP_001180755.1) is presented in Fig 6. The identity of mature human CDNF to marmoset, macaque, and chimpanzee sequences is 93.8%, 100%, and 99.4%, respectively (Table 2). Closely resembling human CDNF and the predicted CDNF sequences of chimpanzee and macaque, marmoset CDNF consists of 187 amino acid (aa) residues with a predicted signal peptide of 26 aa, thus resulting in a mature protein of 161 aa, with eight evolutionarily conserved cysteine residues with identical spacing determining the protein fold [1, 7]. A N-linked glycosylation site of human CDNF at position 57N [13] is lacking from the marmoset CDNF, whereas it is conserved in chimpanzee and rhesus macaque, which, differently from marmoset, belong to apes and Old World monkeys, respectively. However, the *O*-linked glycosylation site at position 181T of human CDNF [54] is conserved in all primate sequences aligned. A putative ER-retention signal KTEL is present at the C-terminal end of human CDNF [55–56] and in the CDNF sequence of chimpanzees and rhesus macaques, whereas in marmoset CDNF, the signal is KAEL where the polar threonine (T) is replaced with a neutral alanine (A) residue.

**Table 2:** Amino acid identity (%) of CDNF proteins between human and selected nonhuman primates calculated by EMBOSS Needle software. Signal peptide sequences have been omitted.

	*Homo sapiens*	*Pan troglodytes*	*Macaca mulatta*	*Callithrix jacchus*
*Homo sapiens*	100	99.4	100	93.8
*Pan troglodytes*		100	99.4	93.2
*Macaca mulatta*			100	93.8
*Callithrix jacchus*				100

## Discussion

### Tolerability of CDNF

The present CDNF chronic infusion study is a first approach in nonhuman primates to define controlled conditions for a safe application for CDNF protein in future therapies.

High-dose chronic infusion of CDNF (15 μg/day for 28 days) led to histopathological changes in two out of four animals. The composition of perivascular infiltrates observed indicated an immunogenicity of recombinant human CDNF in the marmoset brain, presumably caused by interspecies differences. It is also possible that the CDNF protein preparation contained minor impurities inducing the immunological reaction observed. Moreover, we reported inconsistent drug delivery in at least one case. However, the degree of histopathological changes largely matched with the distribution of CDNF-ir, indicating a dose dependency of CDNF immunogenicity. The use of more reliable delivery techniques such as convection-enhanced delivery should be considered for future applications [34–36]. The animal that received a daily concentration of 10 μg CDNF did not show any abnormalities. Although this result is not representative, it is in line with results in rats with a 4–5 times smaller brain where infusion of CDNF at 4.5 μg/day for 2 weeks did not induce histopathological changes [3].

The tolerability of GDNF in marmosets was not investigated in the present study. However, therapeutic doses of GDNF are well tolerated in Parkinsonian marmosets [22, 57]. By contrast, clinical symptoms and histopathological changes can appear after intraputaminal and intraventricular chronic infusion of high doses of GDNF in Old World monkeys [20, 42, 58–59] and PD patients [59]. In particular, meningeal thickening and cerebellar lesions can appear after long-term infusion of high doses of GDNF [42]. Whether CDNF can cause such symptoms (using comparable study parameters) is unclear and warrants further investigation.

Despite the histopathology observed at high and chronic doses, we do recommend CDNF for therapeutic use in marmosets because CDNF is well tolerated in lower concentrations. In the presented treatment approach, the concentration used was more than 20 times less that used in the chronic infusion protocol in which pathological changes occurred. Observations in rodents also confirm that CDNF is well tolerated, even at high therapeutic doses [1, 3]. However, further preclinical studies are needed with CDNF of good-manufacturing-practice (GMP) quality to determine the biosafety of CDNF.

### Neuroregeneration of CDNF and GDNF in the mild 6-OHDA lesion model

In the presented study, we used 6-OHDA to induce moderate to mild caudate lesions in common marmosets, resulting in transient or absent motor dysfunction. However, the impact of the lesion on dopaminergic striatal areas was sufficiently strong to be clearly detected and quantified by ^123^I-FP-CIT SPECT. The SPECT method employed has been previously validated in various studies, and clearly correlated with dopamine dysfunction in models of PD [5, 49, 60–63]. Moreover, this method has gained increasing clinical importance in PD diagnostics [64–65].

In the neurorestorative approach, a significant but moderate increase of DAT binding was observed after treatment with CDNF. Recovery was less prominent than that seen in rodent studies [1–3] and might be explained by the limited regenerative capacity and the larger dimensions of the primate brain compared with rodents. Alternatively, CDNF may work better with a larger lesion and have modest effects with smaller lesions, because it has no effects to the striatum of unlesioned rodent brains [3–4]. From the SPECT data, we cannot distinguish between functional and structural restoration of the dopaminergic innervation. However, previous studies showed a robust correlation of SPECT and TH-ir within the affected areas [60–63]. In comparison with the criterion standard GDNF, CDNF treatment was more effective. This can be explained by the limited diffusion of GDNF compared to CDNF [3, 41] and dose response [3]. More importantly, the differences in the structure and mode of action between CDNF and GDNF may explain the outcome (delayed response of GDNF, long lasting effects of CDNF) [1]. It should be noted that CDNF is structurally unrelated to the classical neurotrophic factors [8] and can rescue neurons when injected into the cell cytoplasm [9]; however, its receptor and signaling pathways remain unclear.

Individual animals responded slightly to GDNF treatment via increased DAT activity, but the effect was statistically not significant. However, at the microscopic level, we found obvious structural effects on TH-ir neurons after treatment with GDNF, but not in marmosets from the CDNF-treated group. The discrepancies between low response in DAT binding and the strong appearance of TH-ir neurons after GDNF treatment are difficult to address. As discussed above, inconsistent drug delivery may account for the low response in the GDNF treatment group [34–36]. Nevertheless, the low impact on DAT activity cannot be explained by insufficient GDNF dose, since the impact of GDNF on TH-ir was very strong. Moreover, we delivered the trophic factors into two injection sites and by that we provided a better distribution of the factors and reduced the risk of insufficient drug delivery. Since only the endpoint of experiment was accessible for histological analysis, only obvious histological changes as mentioned above, may be considered for interpretation. GDNF rather strongly upregulates TH expression and that is one obvious explanation [19]. Possibly, TH-ir striatal neurons do not contribute to DAT activity in a sufficiently strong manner to be detected with SPECT. This possibility is supported by the fact that DAT-ir appeared normal in contrast to TH in the GDNF-treated caudate nucleus. Speculatively, an increase in TH-ir in striatal neurons may compensate for the indistinct dopamine transporter integrity. Whether local interneurons adopted a TH-ir phenotype or whether these cells were newly created remains unclear. Interestingly, striatal dopaminergic neurons were found in PD patients treated with L-DOPA (but not with GDNF), but unlike in the presented study in which marmosets were immunoreactive for TH only, striatal neurons in human PD samples were DAT-ir [66]. This may be explained by differences between humans in non-human primates. Alternatively, striatal TH-ir neurons emerged from effects that were genuine to GDNF-treatment. However, an increase of TH-ir cells is reported in the literature in the context of regeneration and GDNF in Parkinsonian rhesus monkeys [67]. Therefore, we can assume a certain neurorestorative potential also for GDNF, although in a rather limited fashion. By contrast with GDNF, an effect of CDNF on the TH-ir striatal neuron population was not detected in these mildly lesioned animals. Our data support the idea that CDNF and GDNF have different mechanisms of action.

The choice of animal model and treatment approach is essential for testing new therapeutic agents for PD. Neurotrophic factors are strongly neuroprotective, but some of them, such as GDNF, NRTN, CDNF and MANF have also neurorestorative activities [1, 3, 68]. GDNF has shown prominent neurorestorative effects in the primate brain [22, 41]. However, because neurotrophic factor therapies are actually applied to patients at a late stage of disease [37], the presented study focussed on neurorestoration. The 6-OHDA model is considered to induce a robust pathology on DA neurons [49, 69] in which functional regeneration and full anatomical restoration normally does not occur apart from a few exceptions [70–71]. Therefore, the 6-OHDA model is a very good model as it does not mask the effect of the test compound. By contrast, the strong chronicity of the neurotoxin may deplete the endogenous regenerative potential of the brain, and thus prevent the putative beneficial effects of test compounds. In our approach DA neurons in SNc seem not to be affected by the mild impact of striatal lesion as histology performed at the end of experiment may suggest. Taking this into account, the presented mild 6-OHDA lesion protocol effects robust striatal DA neuron pathology, at least on the level of DAT imaging, while maintaining the endogenous regenerative capacity of the brain (in particular the SN), and thus enabling evaluation of the compounds tested. However, since the PBS control group is missing, further investigation is needed to support the suitability and reliability of the proposed lesion paradigm.

In contrast to the viral vector delivered α-syn model of PD, 6-OHDA pathology is restricted to the dopaminergic system. Therefore, it would be interesting to test CDNF in the α-syn model in marmosets [72–74], especially, since GDNF showed no neuroprotective effects in the rat α-syn model [25–26].

### Comparison of marmoset CDNF amino acid sequence within primates

The amino acid sequence of the predicted mature marmoset CDNF is 93.8% identical to that of human CDNF and contains eight cysteine residues of conserved spacing. The predicted CDNF amino acid sequence of our closest living relatives, the chimpanzee, and the rhesus macaque, are even more closely related to the mature human CDNF with an amino acid identity of 99.4% and 100%, respectively. Because of these close homologies, recombinant human CDNF presumably has neuroprotective activities not only in marmosets, but potentially in other nonhuman primates.

Recent publications suggest that human MANF is retained in the ER by the C-terminal RTDL sequence via interaction with Lys-Asp-Glu-Leu (KDEL) endoplasmic reticulum protein retention receptor [75–76]. Whether the C-terminal KTEL sequence in human, chimpanzee, and rhesus macaque or the KAEL sequence in the marmoset CDNF has a similar role remains unclear. Interestingly, an N-linked glycosylation site of human CDNF sequence [13] is also present in the predicted amino acid sequence of the chimpanzee and rhesus monkey, whereas in the sequence of marmoset CDNF, the site is missing. Although glycosylation is not required for the secretion of CDNF [1, 54], the site for N-linked glycosylation may serve some other regulatory function in close human relatives.

### Conclusion

The study demonstrated a beneficial effect of CDNF on DAT activity in a mild 6-OHDA-mediated model of PD in marmoset monkeys. In comparison with CDNF, GDNF treatment was less effective at the level of DAT imaging. However, increased numbers of TH-ir neurons were observed within the lesioned caudate nucleus, indicating the strong regenerative potential of GDNF. In marmosets, intracerebral CDNF treatment was safe when applied at low dose. Chronic delivery of CDNF at a concentration of 15μg/day for 28 days lead to histopathological effects.

## Supporting Information

S1 Fig Photographs of the striatum 60 days after single intracerebral injection of 30μg CDNF. Histological evaluation was based on HE-staining. No pathological abnormalities were observed, with the exception of mild edema at the tip of the needle track (inset, indicated by arrows). Scale bars: overview = 1 mm, inset = 200 μm.
(TIF)

S2 Fig Coronal sections of CDNF-stained marmoset brains after high dose chronic infusion of 15μg CDNF (upper row), 10μg CDNF (lower right) and PBS (lower left). CDNF-ir distribution was restricted to striatal areas, the vicinity of cannula track or absent (target areas indicated by black arrows).
(TIF)

S3 Fig Chronic infusion of CDNF: Body weights remained stable with only transient fluctuations within a range of less than 10%. (TIF)

S4 Fig Quantitative analysis of 123I-FP-CIT SPECT. No spontaneous recovery of DAT activity was observed in the PBS-treated control animal.
(TIF)

S1 Table Density of TH-ir neurons in the substantia nigra pars compacta (SNc). No significant changes were observed between ipsilateral and contralateral SNc of 6-OHDA-lesioned animals after treatment with neurotrophic factors. Moreover, no significant changes were detected between treatment groups. Data are given as mean ± SD.
(DOCX)
