# Supplementary figures and images for: Comparative Analysis of the Effects of Neurotrophic Factors CDNF and GDNF in a Nonhuman Primate Model of Parkinson’s Disease
Source: PLoS One. 2016 Feb 22;11(2):e0149776. doi: 10.1371/journal.pone.0149776 (PMC4763937; doi:10.1371/journal.pone.0149776)

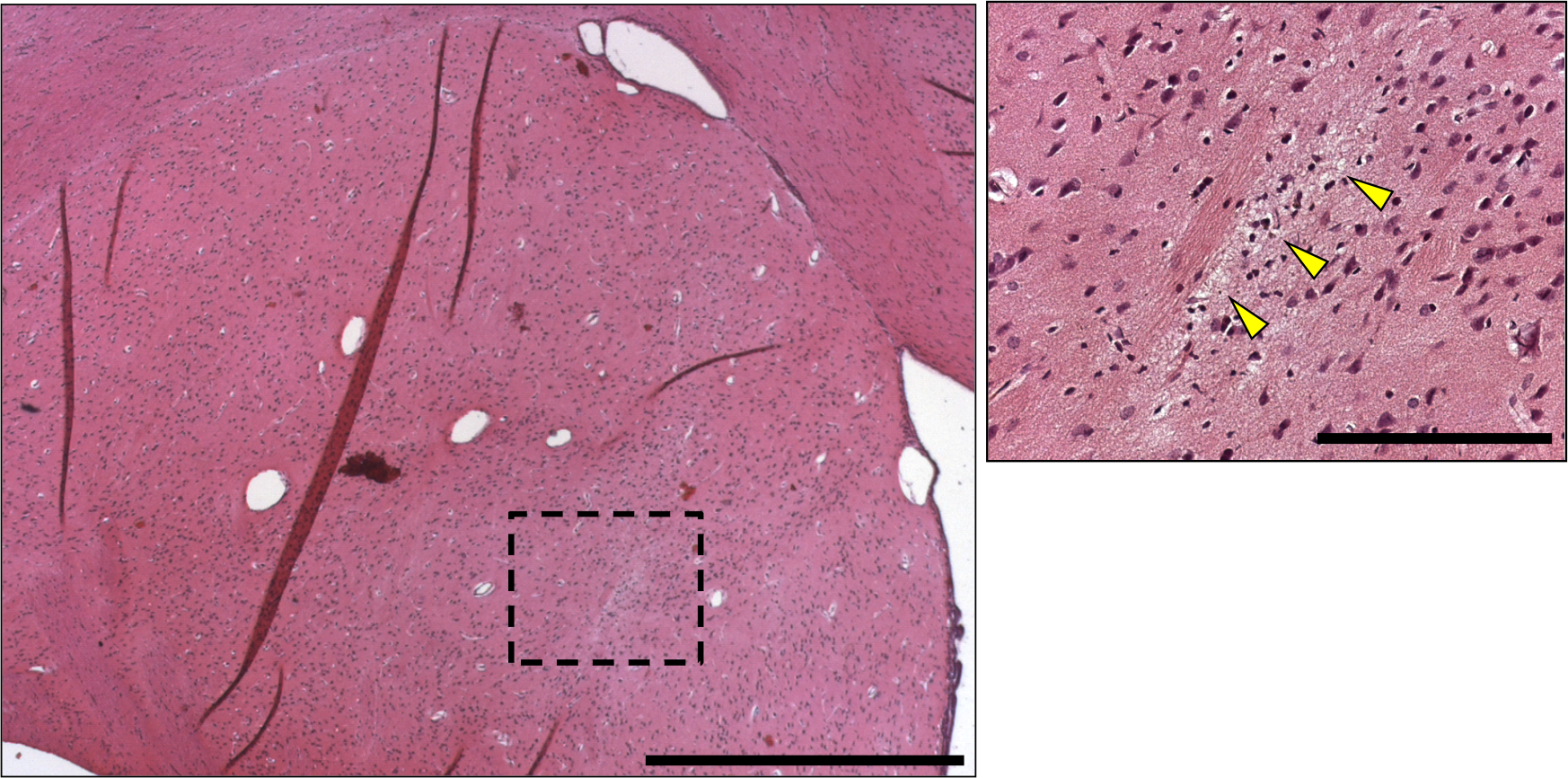

Supplement: S1 Fig — Histological evaluation was based on HE-staining. No pathological abnormalities were observed, with the exception of mild edema at the tip of the needle track (inset, indicated by arrows). Scale bars: overview = 1 mm, inset = 200 μm. (TIF) [file pone.0149776.s001.tif]

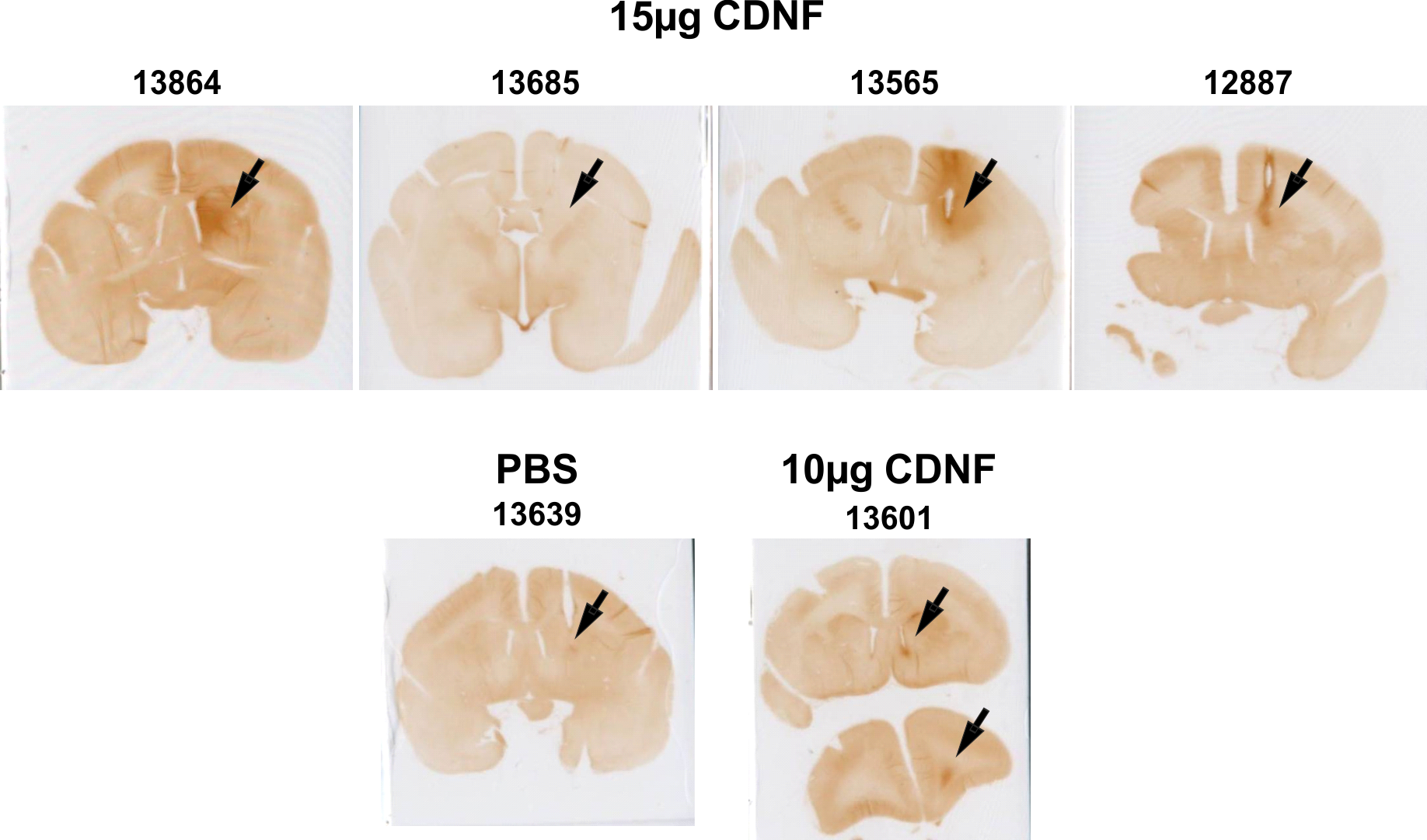

Supplement: S2 Fig — CDNF-ir distribution was restricted to striatal areas, the vicinity of cannula track or absent (target areas indicated by black arrows). (TIF) [file pone.0149776.s002.tif]

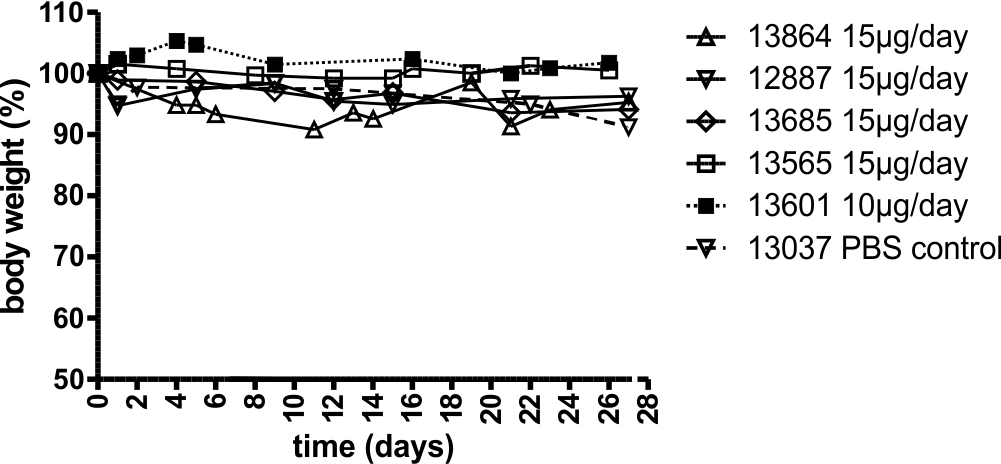

Supplement: S3 Fig — (TIF) [file pone.0149776.s003.tif]

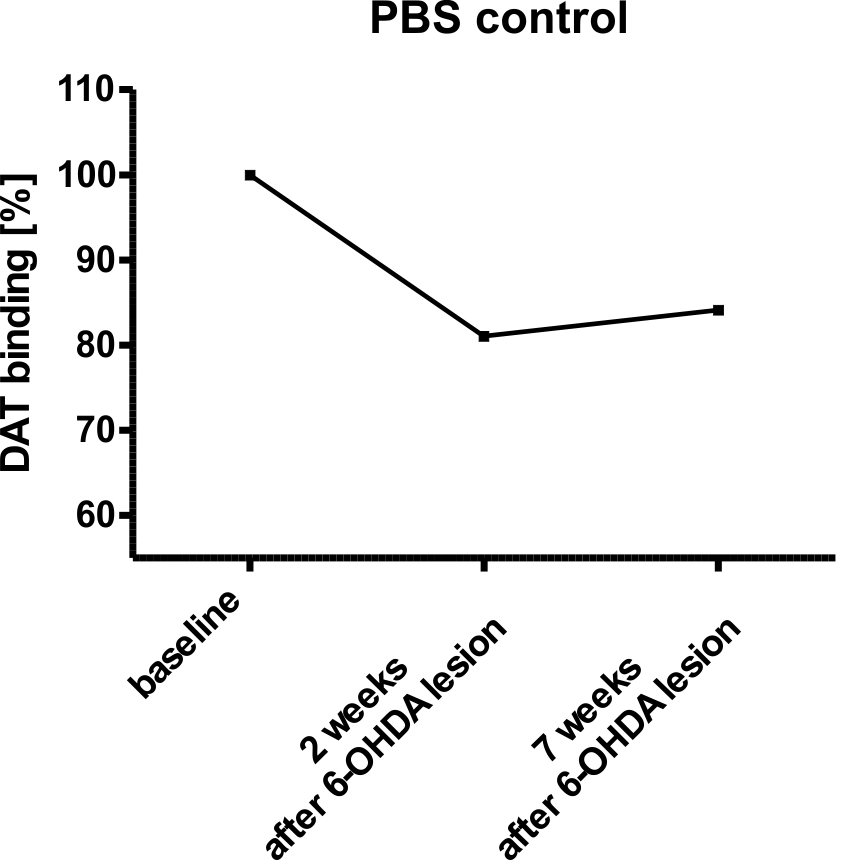

Supplement: S4 Fig — No spontaneous recovery of DAT activity was observed in the PBS-treated control animal. (TIF) [file pone.0149776.s004.tif]
