# Supplementary material for: Comparative Analysis of the Effects of Neurotrophic Factors CDNF and GDNF in a Nonhuman Primate Model of Parkinson’s Disease
Source: PLoS One. 2016 Feb 22;11(2):e0149776. doi: 10.1371/journal.pone.0149776 (PMC4763937; doi:10.1371/journal.pone.0149776)
Supplement: S1 Table — No significant changes were observed between ipsilateral and contralateral SNc of 6-OHDA-lesioned animals after treatment with neurotrophic factors. Moreover, no significant changes were detected between treatment groups. Data are given as mean ± SD. (DOCX) [file pone.0149776.s005.docx]

**S1 Table:**

|  | **TH-ir cells/mm²** | |
| --- | --- | --- |
| **Group** | **SNc lesioned site** | **SNc contralateral site** |
| CDNF (n=4) | 138.50 ± 24.59 | 128.34 ± 28.74 |
| GDNF (n=4) | 126.93 ± 21.54 | 124.78 ± 16.44 |
| PBS (n=1) | 94.98 | 99.63 |
